# Adaptive Discrete Vector Field in Sensor Networks

**DOI:** 10.3390/s18082642

**Published:** 2018-08-12

**Authors:** Mengyi Zhang, Alban Goupil

**Affiliations:** 1 College of Electrical Engineering and Control Science, Nanjing Tech University, Nanjing 210028, China; 2 CReSTIC, University of Reims Champagne-Ardenne, 51100 Reims, France; alban.goupil@univ-reims.fr

**Keywords:** sensor networks, algebraic topology, discrete vector field, discrete Morse theory, distributed algorithm, coverage, sensor selection

## Abstract

Homology groups are a prime tool for measuring the connectivity of a network, and their computation in a distributed and adaptive way is mandatory for their use in sensor networks. In this paper, we propose a solution based on the construction of an adaptive discrete vector field from where, thanks to the discrete Morse theory, the generators of the homology groups are extracted. The efficiency and the adaptability of our approach are tested against two applications: the detection and the localization of the holes in the coverage, and the selection of active sensors ensuring complete coverage.

## 1. Introduction

Many basic issues in the field of sensor networks can be reformulated into a characterization of specific topological spaces [1,2,3]. More precisely, a topological space has a shape that contains a quantity of information that can be recovered by the computation of topological invariants, even if no distance is defined. In addition, the robustness of these topological invariants benefits the applications. As recently highlighted by some researchers—see [4,5] and the references therein—these approaches are worth the effort in many areas ranging from kinematic to data analysis, signal processing, biomedical and genetic engineering.

According to the interpretation of Alexandrov [6,7], the homology groups are effective topological invariants that count the number of independent components, holes, tunnels, etc. It means that the homology groups of a space quantify its connectivity in algebraic terms. This is why they are invariants of choice in the area of sensor networks in which the connectivity issues are of paramount importance.

Our paper contributes to this field by providing a novel distributed algorithm that computes the homology groups. As the context of sensor networks highly affects the algorithm’s design, it must be robust to changes of the network’s topology, such as node extinction, node mobility or emergence of new units. Furthermore, the distribution of the data needs to support these potential changes in the network. Size of the network is another important parameter. Our algorithm can manage different scales of networks from dozens of to thousands of units without unenviable effects. Limiting the communication only between neighbor nodes permits our algorithm to overcome such a challenge.

The solution we propose is based on the concept of discrete vector field introduced in Section 3. This component is an essential ingredient of the discrete Morse theory developed by Forman in [8,9] that links a discrete vector field on a space to its homology groups.

Two applications using our algorithm are discussed at the end of this paper to show the effectiveness of our approach and its robustness. The first one, which is the subject of Section 5, deals with the detection of coverage holes based on the empirically estimated cross-correlations among the sensor measurements. This application was partially published in our previous work [10] but using a centralized and offline homology group computation.

The selection of active sensors is the second application presented in Section 6. Following on from our previous work [11], our algorithm finds a selection of active sensors that guarantee a complete coverage of the area of interest. The other nodes will be turned off in order to save their battery and the lifetime of the network. Our algorithm can reactivate automatically the idle nodes when other nodes shut down.

Other solutions are proposed in the literature to get the generators of the homology groups. Most of them rely on the connection, due to Hodge decomposition, between harmonic forms that are the solutions of a Laplace equation and the homology groups [12,13,14]. This equation is solved by a distributed iterative differential equation solver. This approach works well for homology groups on the real field but not on a finite field as in our setting and, moreover, the convergence analysis of the differential equation solver becomes an issue.

Another approach for computing the homology groups is taken in [15], where the abstract simplicial complex is simplified greedily in a distributed way while maintaining the topology. Once no more simplification is available, the homology groups are computed by a master node. Unfortunately, this solution is not adaptive and the final computation is centralized. However, our solution is conceptually not so far from this approach except that our simplifications are governed by an adaptive discrete vector field and, because of the Morse theory, the generators are readily accessible. Moreover, our proposed solution adapts to changes in the network.

To make this paper self-contained, the next section presents the combinatorial spaces that are of interest as well as its representation as linear spaces. This enables the definition of homology groups. Discrete Morse theory is described in Section 3, which is the backbone of our solution presented in Section 4. The above-mentioned applications are detailed in the two following sections before the conclusion.

## 2. Combinatorial Spaces

The mathematical definition of topological spaces does not provide a convenient representation suitable for automated manipulation. This is why we restrict ourselves to combinatorial spaces that are formed by a finite assembly of basic patches—see [16] for a precise definition of these spaces. The construction of a space and its manipulation are done by managing the assembly instructions. These instructions are provided by the notion of the boundary that maps a patch into the collection of smaller patches that makes up its faces. This mapping maintains all the information about the neighborhood and thus the connectivity associated with the space.

**Figure 1:**
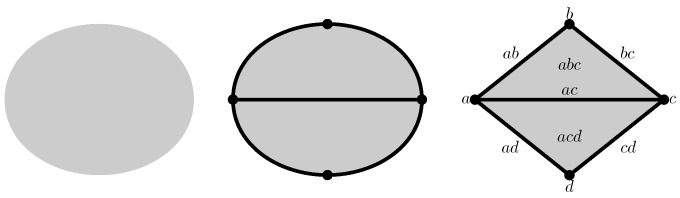
From continuous space to combinatorial space through triangulation.

For example, the space represented on the left of Figure 1 can be triangulated to obtain a topologically equivalent but simpler space drawn in the middle of the figure. The triangulated space is a purely combinatorial construction that brings vertices, edges, and triangles together. Its assembly instructions are provided by the concept of boundaries: the initial space is seen as a combination of two triangles whose boundaries are composed of fives edges whose boundaries are given by four vertices.

### 2.1. Abstract Simplicial Complexes

Simplicial complexes generalize triangulation. The basic elements are not only restricted to vertices, edges, and triangles but include also tetrahedrons and simplexes of higher dimensions. The simplexes are grouped by their dimension. In this way, a *n*-simplex has dimension *n*, has n+1 incident vertices and *n* faces, which are (n−1)-simplexes, and so on.

This construction forces the faces of each simplex to be also parts of the complex. A purely combinatorial description is sufficient to store a space: achieving a spatial realization of a simplicial complex is no longer necessary. We will thereafter focus only on the purely combinatorial approach by using abstract simplicial complexes.

By definition [17], an abstract simplicial complex consists of a finite set *V* of vertices and a family S of subsets of *V*, called simplexes. The collection S should satisfy the closure constraint by subset. That is, if the σ is an element of the family S, then a sub-simplex τ⊂σ is also an element of S. A sub-simplex τ of a simplex σ is called a face of σ. Thus, the closure constraint means that all faces of a simplex are also simplexes.

More precisely, an *n*-simplex σ of dimension *n* is a set of n+1 vertices $\left\{v_{0},v_{1},\ldots ,v_{n}\right\}$. The proper faces are the n−1-simplexes $\left\{v_{0},\ldots ,{\overset{ˇ}{v}}_{i},\ldots ,v_{n}\right\}$, where ${\overset{ˇ}{v}}_{i}$ means that the vertex $v_{i}$ is removed from the list.

The assembly rules of various basic elements given by the simplexes are encoded in the relationship between a simplex and its proper faces. Thus, the boundary of a simplex is given by its proper faces. For example, the boundary of the 2-simplexes {a,b,c}, which is a triangle whose vertices are *a*, *b* and *c*, is composed by its three proper-faces {a,b}, {a,c} and {b,c}, which are obviously its edges.

To make the notations less cluttered, the braces and the commas will often be omitted: the 0-simplex {v} is now identified with the vertex *v* and the triangle {a,b,c} is noted abc, etc. Consequently, the edges of the triangle abc are the edges ab, ac, and bc.

A small generalization of abstract simplicial complexes is provided by regular cell complexes. In this case, the patches used to build a space are not necessarily simplexes. They are vertices, edges, disks, and spheres of all dimensions. Gluing a *d*-dimensional sphere is done along its boundary which is a d−1-dimensional sphere. The cell complex building from its patches stays similar to simplicial complex building, and the boundary operator plays the same role. We will use this small flexibility later in this paper when discussing the Morse theory. In the rest of the paper, the terms *n*-cell and *n*-simplex will be used equally.

### 2.2. Linearization

Introducing the homology groups needs a kind of linearization. This linearization process starts by turning the space *X* into a collection of linear spaces. Once done, the notion of boundary is transformed into a collection of linear maps between the previously mentioned linear spaces.

From now, the vector spaces are defined on the binary field $Z_{2}$. This restriction makes the theory simpler and will not affect the applications that will be presented later. However, if necessary for an application, the extension of our work to other finite fields is still straightforward once the notion of orientation is properly defined.

For each dimension *d*, the vector space $C_{d}$ consists of all linear combinations of *d*-simplexes. The standard basis of $C_{d}$ is given by the collection of *d*-simplexes and, consequently, the dimension of $C_{d}$ is the number of *d*-simplexes of the space. The vectors of $C_{d}$ are called *d*-chains. We won’t note the difference between a simplex σ and the corresponding chains of $C_{d}$.

In the example of Figure 1, $C_{2}$ is a linear space of dimension 2 whose vectors are abc, bcd, abc+bcd, and the zero vector. The space $C_{1}$ is larger: its dimension is 5 and its standard basis is composed of ab, ac, bc, bd and cd. Finally, the standard basis of $C_{0}$ is provided by the four vertices *a*, *b*, *c* and *d*. The others spaces $C_{d}$ are null vector spaces because there is no simplex of dimension *d* greater than 2.

Because of the use of the binary field $Z_{2}$, there is a direct correspondence between *d*-chains and the topological subspaces. For example, the 1-chain ab+bd is associated with the path through the two edges ab and bd.

Once the linear spaces $C_{d}$ are defined, it still remains to link them through boundaries. It is natural to consider that the boundary of the edge ab is given by its vertices *a* and *b*, that is, in equation, $\partial_{1}ab=a+b$. The boundary operator $\partial_{1}$ is defined for all edges in this way. In addition, by linearity, it is also defined for all vectors of $C_{1}$. The intuition behind the boundary of a path is preserved: for example, the boundary of the path from *a* to *d* via the edges ab and bd is $\partial_{1}\left(ab+bd\right)=\partial_{1}ab+\partial_{1}bd=a+b+b+c=a+d$ because, in the binary field, b+b=0. In other words, the boundary of a path between two vertices given by a sum of these vertices.

For the other dimensions, the boundary operator is defined in the same way. For example, $\partial_{2}abc=ab+bc+ac$ and $\partial_{2}bcd=bc+cd+bd$. The boundary of the complete surface of Figure 1 is given by the sum of both: $\partial_{2}\left(abc+bcd\right)=\left(ab+bc+ac\right)+\left(bc+cd+bd\right)=ab+bd+cd+ac$. Of course, the vertices do not have boundaries and therefore $\partial_{0}=0$.

The set of vector spaces and their associated boundary operators are summarized in the following Equation (1), which is the algebraic counterpart of the combinatorial topological space. This sequence of vector spaces and linear operators is called the chain complex, which is noted as $C_{•}$:

(1) $$0\overset{\partial_{n+1}}{\to }C_{n}\overset{\partial_{n}}{\to }C_{n-1}\overset{\partial_{n-1}}{\to }\cdots \overset{\partial_{2}}{\to }C_{1}\overset{\partial_{1}}{\to }C_{0}\overset{\partial_{0}}{\to }0.$$

The most important property of the boundary operators is that $\partial_{d}\partial_{d+1}=0$, which means that the boundary of the boundary is void. Without the indices, this constraint is synthetically written $\partial^{2}=0$. The indexing of the boundary maps $\partial_{d}$ by *d* will often be omitted below; the context will make it clear.

### 2.3. Homology

Homology groups $H_{k}\left(X\right)$ measure the connectivity of the topological space *X*. From them, the notion of *k*-dimensional “holes” or “voids” can be quantified. Algebraically, it is a question of finding the boundary-less *k*-chains around these holes, but, in order to avoid double counting of the same hole, an equivalence relation between *k*-chains needs to be set up properly.

**Figure 2:**
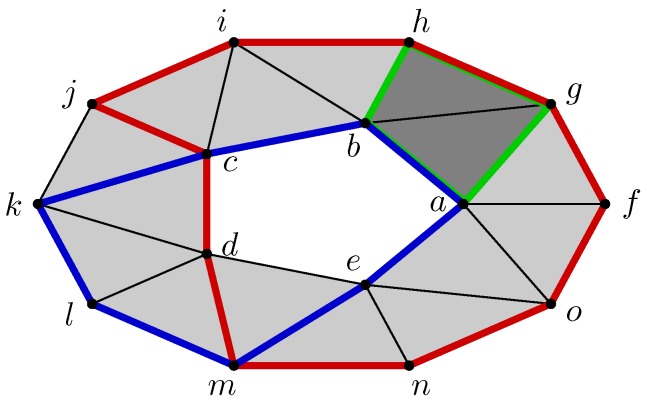
Different kind of cycles: a blue cycle is relevant and span the homology group $H_{1}$; a green cycle is irrelevant because it surrounds no hole as it can be collapsed; a red cycle is equivalent to the blue cycle as it can be transformed into it while staying in the ring.

First of all, boundary-less *k*-chains, or *k*-cycles, are vectors σ of $C_{k}$ such that $\partial_{k}\sigma =0$. For example, the 2-chain ab+bc of Figure 2 is not a cycle because its boundary a+c is not zero. However, the highlighted 2-chain ab+bh+hg+ag is a cycle because its boundary is zero. Intuitively, a cycle encloses a part of the space.

Continuing with the same example, we remark that the blue and red cycles surround the same hole, but it is intuitively possible to “transform continuously” the first cycle in the second while remaining in the space. Similarly, the green cycle does not surround a hole and is equivalent to a zero cycle because it can be “reduced continuously to a point.”

Algebraically, this intuitive equivalence is formulated by saying that two *k*-chains are equivalent if there is a k+1-chain whose boundary is given by the two *k*-chains. Finally, the homology group $H_{k}$ is the linear space that gathers all cycles together identifying two cycles that are equivalent. Mathematically, it corresponds to the quotient of linear subspaces

(2) $$H_{k}\left(C_{•}\right)=\frac{\ker\partial_{k}}{\mathrm{im}\partial_{k+1}}.$$

The dimension of the vector space $H_{0}$ gives the number of connected components of the space; the dimension of $H_{1}$ returns the number of “holes”, the dimension of $H_{2}$ means the number of “voids”, etc. The dimensions $\beta_{0},\ldots ,\beta_{2}$ of $H_{0},\ldots ,H_{2}$ are called the Betti numbers that provide a number of space invariants of prime interest.

Since the boundary operators $\partial_{k}$ are linear operators, it is simple to compute the homology groups $H_{k}$ through matrix reduction when the dimensions of $C_{k}$ are finite. However, this requires a centralized calculation that is not well-suited for sensor networks’ application.

### 2.4. Distributed Data Structure

The design of a data structure in order to handle abstract simplicial complex does not present major difficulties when it is centralized. However, finding a distributed data structure to deal with the simplicial complex is not immediate because of the costs of data replication and communication. The paper [15] proposed a detailed solution that we now summarize.

The basic idea of the data structure is that a node takes care only about the vertex associated with it and only some of the edges, triangles, and general *n*-simplexes containing this vertex.

More precisely, each vertex is identifiable by its id that is sortable such as the underlying sensor media access control address (MAC address). A simplex σ is managed by only two nodes that have the smallest id among the vertex of σ. In this way, adding or removing a simplex in the data structure involves agreements between only two nodes. In addition, two sensors managing the same simplex are often nearby and thus the burden of communication is reduced.

**Figure 3:**
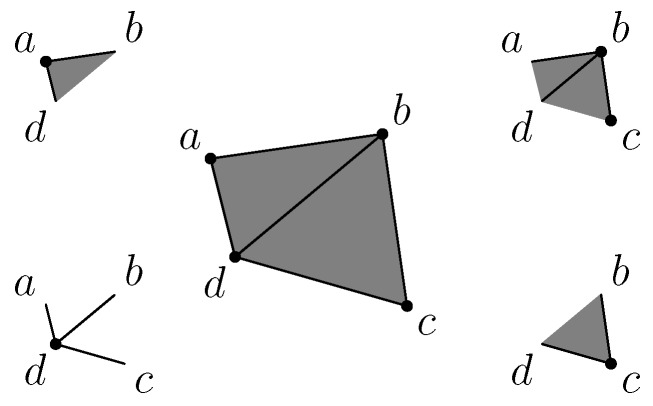
Distributed data structure representing the simplicial complex shown in the middle.

For example, the data structure of the abstract simplicial complex at the center of Figure 3 is distributed to the nodes *a*, *b*, *c* and *d* according to the schemes displayed in the corner of this figure.

Each node manages its vertex and all edges adjacent to itself. For example, the sensor *a* stores and manages the vertex *a* and the edges ab and ad. It has no knowledge of the edges bc, cd and bd. This part of the data structure is equivalent to a distributed storage of a graph. The difference appears with simplexes of higher dimensions. For example, the triangle bcd is only managed by the sensors *b* and *c*. In addition, sensor *d* is not aware of this triangle. The vertex *c* manages simplexes *c*, bc, cd and bcd, while the vertex *d* takes care only of simplexes *d*, ad, bd and cd, as shown in the lower right corner and the lower left corner of the Figure 3, respectively.

Keeping this distributed data structure coherent is simplified because the replication factor is 2 and the manager of a given simplex is carried out by two neighbor sensors: addition or removal of a simplex requires the two nodes of the smallest ids of this simplex to modify their own storage. No other sensor takes part in these requests.

## 3. Discrete Morse Theory

A direct computation of the homology groups is not conceivable in a distributed and constrained environment. Hopefully, homology groups are not changed if the space is neatly reduced. Based on the discrete Morse theory, our algorithm reduces the space thanks to a discrete vector field and the output of the algorithm can be read directly without further processing. Moreover, our approach puts up nicely with the variation of the underlying space, such as deletions or additions of simplexes.

The discrete Morse theory, as its name suggests, is the discrete counterpart developed by Forman [8,9] of the continuous Morse theory [18]. The basic idea is to define a vector field that specifies how to reduce a space while retaining its homology. We present the discrete version of the Morse theory in this section, starting with the vector fields and the connection they have with the homology groups.

Discrete Morse theory was initially thought for cellular complex, which is for space defined by cells. However, as simplicial complexes are a special kind of cell complex, the theory can be applied without any difficulty.

### 3.1. Discrete Vector Field

A discrete vector on a simplicial complex *X* is a pairing (σ,τ) between a simplex σ and one of its proper co-faces τ, such as between a vertex and an incident edge or between an edge and an incident triangle, etc. A vector (σ,τ) is displayed in the figures of this paper by an arrow from the middle of σ to the middle of τ. A discrete vector field is a collection of discrete vectors with the constraint that a simplex is involved in at most one vector. A simplex is called paired if it is a component of vector field, otherwise it is called critical.

**Figure 4:**
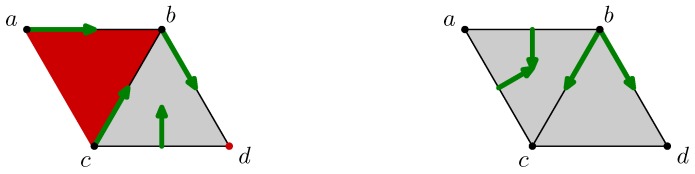
Discrete vector field on the right. On the left, the vectors do not form a field.

Some illustrations of the previous concepts are given in Figure 4. A simplicial complex and a discrete vector field are shown in the right part. The red simplexes are the critical cells: the vertex *d*, the edge ac and the triangle abc. On the right side of the Figure 4, a set of vectors is also drawn on the same simplicial complex, but they do not form a vector field because the two vectors (ab,abc) and (ac,abc) point to the same cell, and the vectors (b,bc) and (b,bd) have the same cell as a tail. Thus, the two cells abc and *b* are involved in several vectors, which is not allowed by definition.

A vector field defines a new linear operator *V* on the chains whose action is defined by the values of *V* on the standard basis. The value Vσ of a *k*-cell σ is given according to its class. If σ is the tail of a vector (σ,τ) where τ is a k+1-cell, then Vσ=τ; otherwise, Vσ=0. The definition of the vector field guarantees that *V* is well-defined.

Looking back to the left side of Figure 4, we have Va=ab, V(b+c)=Vb+Vc=bd+bc and V(ac)=0. In the same way, V(cd+bc)=V(cd)+V(bc)=bcd+0=bcd.

Note that a discrete vector involves only two incident cells. Thus, its distributed storage management does not demand a specific data structure; an add-on of the data structure storing the simplicial complex is enough.

### 3.2. Flow from Discrete Vector Field

As in the continuous case, a discrete vector field and its associated operator define a flow on the *k*-chains. It combines a natural motion from cells of higher dimension to the ones of lower dimension and a motion dictated by the vector of the discrete vector field: the flow Φ is a linear operator on *k*-chains driven by the vector field *V* according to

(3) Φσ=σ+V∂σ+∂Vσ.

Consider a few examples in order to understand the behavior of this flow operator. Consider, once again, the discrete vector field on the left side of Figure 4. Here, Φ(a)=a+∂Va+V∂a=a+∂(ab)+0=a+a+b=b. Actually, the vertex *a* seems to move to *b* according to the discrete vector field. The next example illustrates the cumulative effects between the part of the flow passing through the k−1 dimension, i.e., V∂ and the one passing through the k+1 dimension, i.e., ∂V. The simplex (cd) moves to Φ(cd)=cd+∂V(cd)+V∂(cd)=cd+∂(bcd)+V(c+d)=cd+(bc+cd+bd)+(bc+0)=bd. Intuitively, the edge cd moves to bd and bc, but, as the vertex *c* also moves to *b*, the edge bc is not taken into account, and there is a simultaneous displacement of the edge and of a vertex of this edge.

Distributed computation of the displacement generated by the flow Φ is particularly simple to implement because, by its definition (3), it involves only purely local operations. Indeed, a cell of a chain is moved to another incident cell according to vectors composed of local cells. Thus, the calculation of Φ is equivalent to the local spread of messages led by the discrete vector field.

### 3.3. Morse Decomposition

By repeating the movement according to the flow Φ, a dynamic system can be identified through the iterated application $\Phi^{k}\left(\sigma \right)=\Phi \left(\Phi^{k-1}\left(\sigma \right)\right)$ and $\Phi^{0}\left(\sigma \right)=\sigma$. As in our case, the complex is finite. Starting from a chain *c*, two asymptotic behaviors of $\Phi^{k}\left(c\right)$ can occur: either a fixed point is reached, or a periodic phenomenon takes place.

As a first step, we assume that only fixed points exist that solve the equation $\Phi^{\infty }\left(x\right)=x$. Then, a cell complex, called a Morse complex, which has exactly the same homology groups as the initial space, can be built. By construction, the cells of the Morse complex are shadows of the critical cells of the discrete vector field. Boundary operators of the Morse complex are supplied by following the flow Φ.

**Figure 5:**
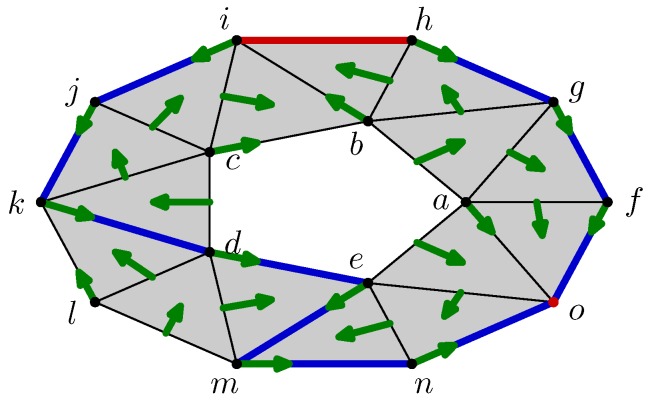
Example of a discrete vector field on the ring.

**Figure 6:**
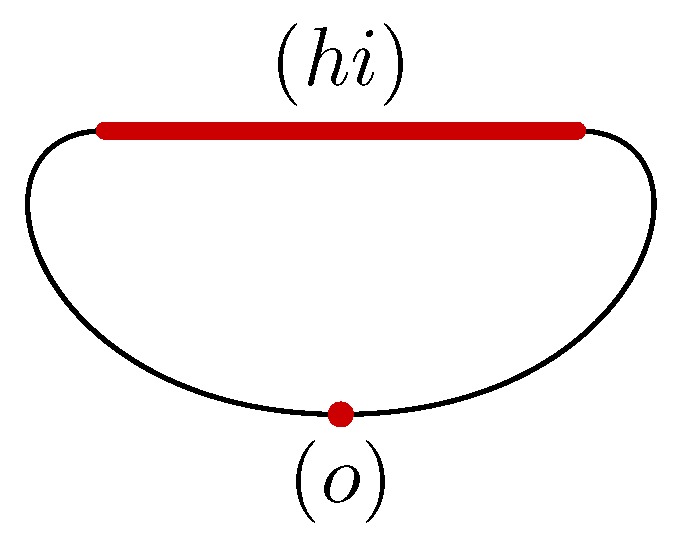
Morse complex of the ring from the discrete vector field of Figure 5.

An illustration of this construction is provided by considering the triangulated torus of Figure 5, which is the same as the space of Figure 2 but annotated by a discrete vector field *V*. In this case, two cells are critical: the edge hi and the vertex *o*. The Morse complex associated with the torus and its discrete vector field is shown in Figure 6. It is composed of the shadow cells (hi) and (o) of dimension 1 and 0, respectively, where the parentheses distinguish the critical cells of initial space from the cells of Morse complex.

It remains to link these two cells (hi) and (o) by the boundary operator. For fulfilling this task, it suffices to follow the flow starting from the boundary of hi, which are the vertices *h* and *i*. As $\Phi^{\infty }\left(h\right)=o$ and $\Phi^{\infty }\left(i\right)=o$ as well, the boundary of the cell (hi) is (o), which is counted twice as shown in Figure 6.

The theory developed by Forman in [8] asserts the equivalence, in the sense of isomorphism, of the homology groups between the original space and the Morse complex built from a coherent discrete vector field. Indeed, the homology of a torus is the same as the homology of a circle. In addition, as the homology groups are isomorphic, it is possible to build a correspondence between the generators of the homology group of the original complex and the generators of the homology group of the Morse complex.

In the previous example, (hi) is a generator of the homology group $H_{1}$ of the Morse complex. It is matched with the cycle hi+ij+kd+de+em+mn+no+gh+fg+fo highlighted in blue in the Figure 5. This cycle can be reconstructed from the critical cell hi by following the edges crossed during the flow iteration $\Phi^{k}\left(hi\right)$.

From the homological point of view, there is no difference between the initial simplicial complex and the Morse complex because it is possible to match in one-to-one way generators from one complex to the other one. Moreover, these matchings between generators are straightforward to perform because they are based on the flow of a discrete vector field. The distributed implementation in a network is practically done via a simple exchange of messages between nearby nodes.

The Morse complex approach for the distributed computation of the homology group led to a drastic reduction of the size of the problem. Even in our simple example, instead of an area consisting of 15 triangles, 30 edges and 15 vertices, the homologically equivalent Morse complex results in a single edge and a single vertex.

Undeniably, the effectiveness of this approach depends heavily on the construction of the discrete vector field. Empirically, a random construction turns out to be optimal most of the time [19]. This means that, with high probability, an edge of Morse complex built with the help of a random discrete vector field corresponds directly with a generator of $H_{1}$, which means a hole. If the optimality is not reached, it should be close.

Whatever the optimality of the Morse complex is, the number of holes is necessarily bounded above by the number of critical edges. From our experiments, this bound is very tight. In addition, the construction of a cycle surrounding a hole is made by following the flow that starts from critical edges. This process is further rerun as soon as other critical edges are met.

## 4. Adaptive Discrete Vector Field

As was shown in the previous section, the discrete Morse theory can be used to compute the homology groups of a combinatorial space through a discrete vector field. Moreover, there is a close correspondence between the generators of the homology group $H_{k}$ and the critical *k*-cells. Thus, it is sufficient to manage a discrete vector field cleverly in order to quickly get all the relevant homological information of the underlying combinatorial space.

We provide in this section an algorithm that updates a discrete vector field when the underlying combinatorial space undergoes changes, such as addition or removal of a collection of simplexes.

However, once again, as vectors are local data involving only two nearby simplexes, the proposed updating procedure can be carried out in parallel in many places without any coordination.

The initial discrete vector field can be constructed randomly according to the procedure described and studied in [19]. Another solution for the initialization consists of starting with an initially empty field and then complete it step by step according to the algorithm described below.

We would like to also point out that the homology group being a quotient space, our solution gives only a representative for each generator. This representative is not necessarily optimal for a certain criterion.

### 4.1. Discrete Vector Field Updating

The updating procedures are based on the same principle for insertion of a set of cells or their withdrawals. First, a collection of relevant simplexes is gathered. Afterwards, vectors are added between simplexes of this collection in a greedy way as far as possible.

In order to render the presentation below easier, we introduce some terminologies. During the updating, the state of a simplex can be

- *matched* meaning that the simplex is involved in a discrete vector;
- *critical* indicating that the cell is critical according to the current discrete vector field;
- *unsettled* for cells whose status is not yet defined precisely during the update.

Naturally, the *unsettled* state is a transient state for simplex. The other two states, *matched* and *critical*, depend on the discrete vector field, which means that no change is scheduled for now.

Once labeled, the *unsettled* cells are going to create new vectors according to the greedy Algorithm 1, which is similar to the one presented in [19]. At the end of the algorithm, the simplexes are either matched or critical, but no one remains *unsettled*.

**Algorithm 1:** Greedy matching by discrete vector.  
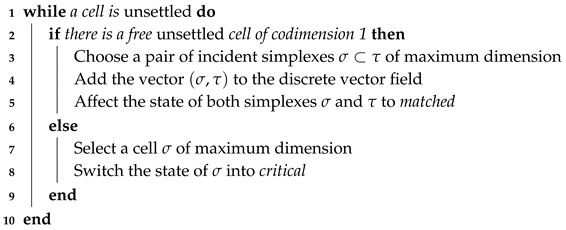


The statements of this algorithm modify only locally the data by modifying the vector and simplexes close to each other. Therefore, the data structures are updated only by exchanging messages between neighbor nodes who manage *unsettled* cells.

#### 4.1.1. Update for Insertion

When cells are added to the abstract simplicial complex, they are initially labeled *unsettled*. The critical cells that are on the boundary of *unsettled* cells are also labeled *unsettled*. Once all *unsettled* cells are correctly labeled, Algorithm 1 will run.

**Figure 7:**
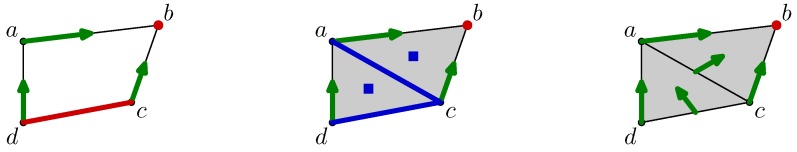
Discrete vector field update while inserting simplices.

Figure 7 depicts the filling of the interior of the quadrilateral abcd when the triangles abc, acd, and the edge ac are added. The states of these three cells are *unsettled* as shown by blue fat edges or a small square inscribed in them. However, as the edge cd was previously critical and is on the boundary of the *unsettled* triangle acd, its state is turned into *unsettled*.

The center of Figure 7 shows the states of all cells before the matching algorithm. Notice that the state of the critical vertex *b* is not modified because it is not on the boundary of any *unsettled* cells.

Finally, Algorithm 1 runs and matches in a greedy way the *unsettled* cells. The result is given in the right part of Figure 7 in which the discrete vector field is optimal.

#### 4.1.2. Update for Removal

Removal of cells is motivated by the same spirit of simplification. The cells that were matched with one of the removed cells are labeled *unsettled* as well as the critical cells on the boundary of *unsettled* cells. The discrete vector field is then rebuilt by Algorithm 1.

**Figure 8:**
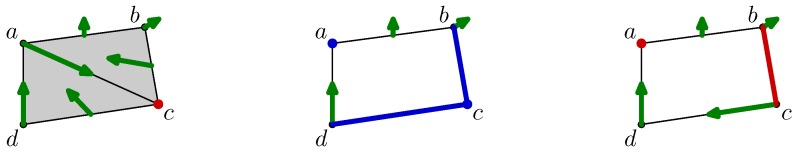
Discrete vector field update while removing simplices.

For example, the three simplexes abc, acd and ac will be removed from the full quadrilateral abcd drawn on the left of the Figure 8. Once it is done, the state of the simplexes *a*, bc and cd switches to *unsettled* because they were matched by the vector field with removed cells. Moreover, the state of the critical vertex *c*, as on the boundary of the *unsettled* edge bc, switches as well. The result of this updating step is shown in the middle part of the Figure 8, in which, as previously, the blue edges and the vertices point represents the *unsettled* cells.

As for insertion update, Algorithm 1 is run to settle the state of all cells. The right of Figure 8 shows a possible solution found.

### 4.2. Vector Field Simplification and Computational Optimization

As the discrete vectors are added or removed locally without trying to optimize the global discrete vector field, performing regularly global simplifications may be beneficial. In his papers [8,9], Forman introduces this kind of simplification, which is called canceling critical points. The continuous part of the Morse theory also has an equivalent canceling [18].

The canceling simplification is possible if there is a path following the discrete vector field between two critical cells. If this path is unique, then the vector can be “reversed” smoothly. As a consequence, the number of critical cells and the size of the Morse complex are reduced by two.

**Figure 9:**
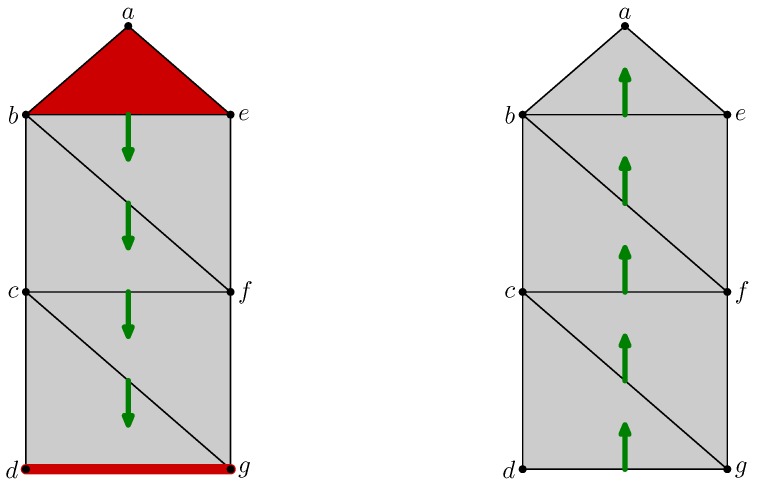
Discrete vector field simplification.

Figure 9 shows the operation on the same example as Forman’s [8]. On the left, there is a path from the critical triangle abe to the critical edge dg. Assuming that this path is unique, the vector can be changed such as in the right part of the figure. The number of vectors increases, but the two critical cells disappear. The uniqueness of the path avoids the risk of creating periodic orbit in the dynamical system defined by the flow.

The simplification operation can be applied in a distributed and asynchronous way. It suffices to follow the path according to the discrete vector field from each critical cell and, once a unique path is found toward another critical cell, the reversion is performed by going backward along the founded path.

These simplifications are not essential as they do not change the result of our approach. Thus, their scheduling depends only on the application. In all cases, we recommend performing them at a small rate because the simplifications make the further computations of cycle generators of the homology groups faster.

In order to compute the *k*-th homology group, it is not necessary to store the complete simplicial complex. It is possible to limit the storage to its k+1-th skeleton: all cells of dimension greater than k+1 may be ignored. Our solution will not provide the same vector field in both cases, but the Morse theory gives analogous decomposition. As the size of the simplicial complex depends locally on the degree of the vertices, this limitation may be welcome.

### 4.3. Comparison with Other Algorithms

In [15], the authors describe a distributed algorithm to compute the homology groups of a simplicial complex stored locally in the network. Unlike our solution, their algorithm performs local simplification through reductions and co-reductions of the abstract complex to obtain a thinner complex representation. This output is then sent to a master node, which completes the computation of the homology by a classical algorithm such as the one presented in [17].

The adaptability of the method in [15] is limited because of the use of reductions and co-reductions that must be recomputed with each change of the simplicial complex. Our solution does not suffer from this defect thanks to the discrete vector field, which encodes reductions and co-reductions in a very flexible way.

The approach in [20] as well as in the associated paper [21] is based on zigzag persistent homology [22], which is also different from our algorithm. This approach requires a complete knowledge of the history of the construction of the complex prior to its analysis. Thus, it is not suitable for the situation of an adaptive algorithm. Moreover, zigzag persistence needs to merge the abstract complexes given at two distinct instants, which asks for a fine management of the data structure, especially if the distribution of the algorithm is considered. Finally, we are not aware of a distributed algorithm for the computation of zigzag persistent homology.

## 5. Covering Application

The first application of our algorithm deals with the detection of holes in the coverage of a wireless sensor network. This detection is based on the results of [23,24] that relate the holes in the coverage and the dimension of homology group $H_{1}$ of an abstract simplicial complex that approximates, in a coarse sense, the coverage. This approach is especially powerful as it does not require the knowledge of sensor positions. The relationship between the measuring field and the geography is thinly encoded through an inequality between the covering radius and the communication radius of each sensor.

Several implementations of this topological approach have been proposed, such as in [25], where the problem is reformulated as a distributed optimization program, or in [26], which uses the isomorphism between the homology groups and harmonic functions on a graph, or in [15], which simplifies with a distributed algorithm the spatial approximation before the computation of an equivalent homology group at a master node of the network. The authors of the paper [14] propose also a distributed algorithm based on harmonic form computation and topological persistence to detect and localize coverage holes. Their solution recovers tight generators around holes and, consequently, the localization is easier. Our solution is not necessarily optimal in the sense of tightness of the generator founded. A second step of optimization of the length of the generator is needed if the application requires it. Several approaches are possible such as in [27] or [28] for a completely topological solution. However, our algorithm is adaptive and changes in the network topology do not modify dramatically the proposed generators. This aspect is often needed to provide a certain robustness of the application, especially during the transient phase of the network construction.

In a previous work [10], a suitable notion of coverage was introduced using a Gaussian Process model where a “hole” is defined as a location where the prediction of the field of interest from the measurements of the network is not precise enough. The prediction $f_{*}$ at a location $x_{*}$ from the measurements by the sensors *z* follows a normal law whose expression is [29]

(4) $$f_{*}|z∼N\left(K_{*}\;{\left(K+\sigma^{2}I\right)}^{-1}\;z,K_{**}-K_{*}\;{\left(K+\sigma^{2}I\right)}^{-1}\;K_{*}^{T}\right).$$

The variance of the prediction depends only on the correlations between sensor nodes’ measurements *K* and between sensor nodes and location $x_{*}$, $K_{*}$.

The paper [10] describes a simplicial complex that approximates the space where the variance of the prediction is smaller than a given threshold. The main point is that the location of the sensors is not necessary to know and this approximation is only based on the correlations between sensor nodes.

However, the approach in [10] is static since it requires the correlations are given at the beginning and the approximation construction is done afterwards. We propose removing this constraint using the algorithms of Section 4 and online estimation. For example, when the sensor *s* collects a new measurement $z_{s}$, it updates the estimated mean ${\bar{z}}_{s}\left(k\right)$ at instant *k* by a low pass filter

(5) $${\bar{z}}_{s}\left(k\right)={\bar{z}}_{s}\left(k-1\right)+\alpha \left(z_{s}-{\bar{z}}_{s}\left(k-1\right)\right),$$

where α is a factor close to 0. The correlation $\sigma_{st}\left(k\right)$ at time *k* between the sensors *s* and *t* is also updated similarly

(6) $$\sigma_{st}\left(k\right)=\sigma_{st}\left(k-1\right)+\beta \left(\sigma_{st}\left(k-1\right)+\left(z_{s}-{\bar{z}}_{s}\left(k\right)\right)\left(z_{t}-{\bar{z}}_{t}\left(k\right)\right)\right),$$

where β is another factor just like α.

Using the correlation estimations $\sigma_{st}\left(k\right)$ instead of the exact correlation, an approximation of the coverage can be obtained in the form of a simplicial complex as described in [10]. Of course, as the time flies, the approximating space will change. Initially, this simplicial complex is only made up of vertices, one for each sensor. It is updated as soon as the updated cross-correlations modify the edges of the construction. In parallel, a discrete vector field, which is initially empty, is modified according to the procedure described in Section 4 each time an edge, or a triangle appears or disappears.

**Figure 10:**
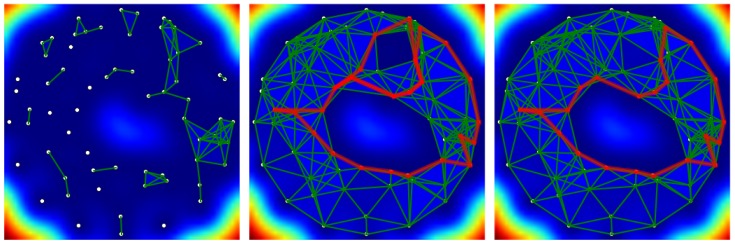
Detection of holes in the coverage by online correlation estimation.

Figure 10 shows the evolution of the detection of coverage holes by an example. The white dots indicate the position of the sensors, while the colors of the background indicate the theoretical precision of the prediction of the field of interest. The sensors are randomly placed on an annulus in order to create a hole artificially, as the light blue indicates at the center of the figure.

At the beginning of the simulation, in the left part of the figure, the simplicial complex holds only a few edges because the estimation of correlations is still too poor to identify viable edges. After a certain delay, as shown in the center of the figure, the simplicial complex approaches the coverage in a satisfactory way. Thanks to the discrete vector field, two holes are detected and localized by the two generators of the homology group $H_{1}$, which are visualized by red paths in Figure 10.

The discrete Morse theory permits tracking the coverage holes and all changes of the approximating complex. Note that this approach is stable in the sense that a local modification of the complex does not modify a cycle generator $H_{1}$ too deeply. For example, the complex on the right of the Figure 10 obtained after the arrival of a single edge inside the small hole at the top which is due to a better estimation of the correlation, will remove a hole in the coverage, but the extinction of this hole does not affect profoundly the cycle around the main hole. This property is important because it allows a simplified monitoring of the holes by the operator. As a disadvantage, the generators are not necessarily close to the holes and may even surround several ones. A second optimization step is needed if tight generators are required as explained previously.

In summary, the application of discrete Morse theory with the proposed updating algorithms permits detecting and tracking, in real time and in a distributed way, the holes of coverage which are defined as the areas of the field that may not be estimated well enough in sensor networks. This application is only based on the cross-correlation estimation between the sensors and does not require the position of the sensors or meta-parameters of an underlying mathematical model. In addition, our updating algorithms allow us to follow the evolution of the coverage at lower costs.

## 6. Selection Application

The second application of our method based on the adaptive discrete Morse theory deals with the second part of the paper of de Silva and Ghrist [24]. If the holes of coverage are detected by the computation of the homology group $H_{1}$, a sufficient subset of sensors that guarantee the coverage is accessible via the homology group $H_{2}$.

More specifically, let *X* be the simplicial complex that represents the coverage. The fence of the covered area is marked out by the sensors set *F* as well as the edges between them. As discussed previously, if $H_{1}\left(X\right)$ is trivial, there is no hole in the coverage of *X*. However, in this case, $H_{2}\left(X,F\right)$ contains a single generator that is an algebraic sum of triangles whose vertices are sufficient to cover the area *X*. The other vertices can be put in a sleeping mode in order to save their battery without any risk to tear the coverage. However, if $H_{1}\left(X\right)$ is non-trivial, indicating a hole in the coverage, $H_{2}\left(X,F\right)$ becomes trivial. Thus, H1(X) and $H_{2}\left(X,F\right)$ provide both a hole detector.

Before explaining the algorithms used in this application in Section 6.2, we first describe a simulation setup and the results provided by our algorithms. The following subsections deal with the details of using the discrete vector field for sensors’ activation application.

### 6.1. Simulation Example

**Figure 11:**
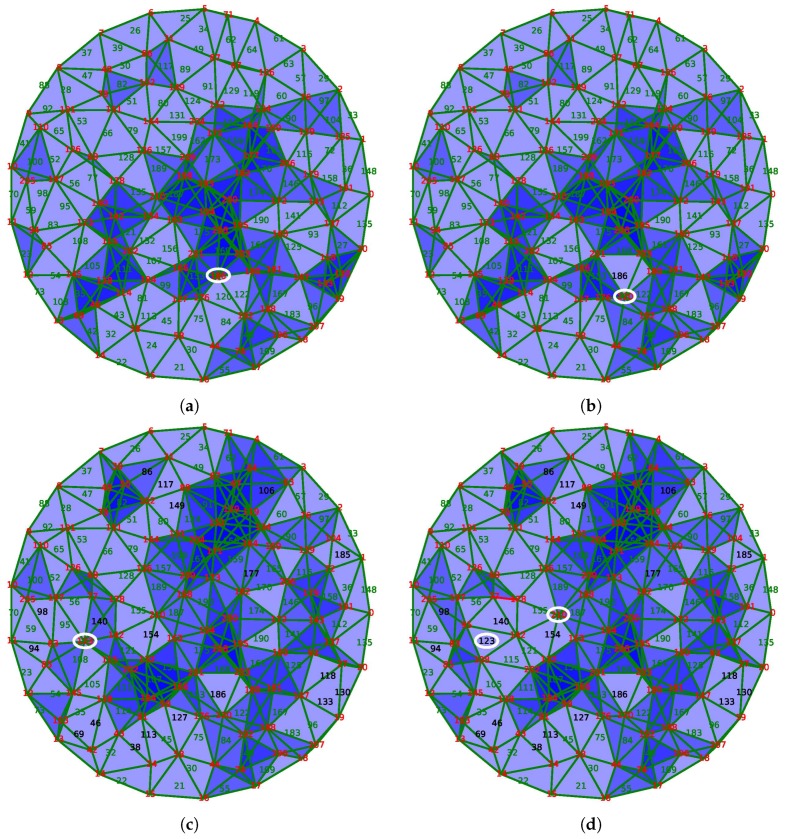

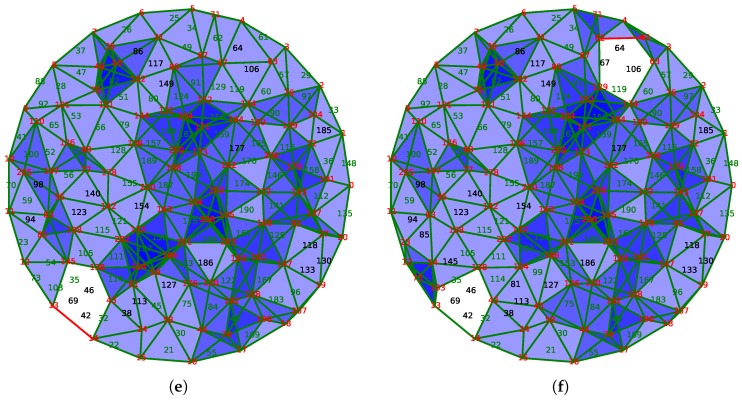
Snapshots of awoken and asleep sensors at iteration number (**a**) 1; (**b**) 2; (**c**) 20; (**d**) 21; (**e**) 23 and (**f**) 27.

Figure 11 illustrates the process of the application as a function of time. Twenty sensors are placed uniformly along the boundary *F* of a disk. Each sensor is connected to its two nearest neighbors by an edge. We assume that these sensors are active throughout the life of the network.

The sampling algorithm described in [30] is used to put 190 sensors uniformly inside the disk. In the figure, the sensors are identified by a number whose color indicates the status. Red numbers indicate active sensors, whereas green numbers show the sensors in sleeping mode. The black color is used for dead sensors by lack of energy.

Two sensors are connected by an edge if the distance between them is less than a fixed radius. The simplicial complex *X* approximating the coverage is the clique complex constructed from the graph of connections as in [24]. In this paper, the authors prove that, under a hypothesis between the communication radius and the sensing radius, this simplicial complex represents the coverage adequately. In Figure 11, the covered triangles are drawn in blue with a certain transparency. In this way, the depth of the color indicates the redundancy of the coverage using the awoken nodes only.

Finally, an initial discrete random vector field is built on the complex by the method of [19]. It means that we assume that the sensor network covers perfectly the area at the beginning.

Initially, the sensors receive a random reserve of energy, which exhausts in time according to a rate which is higher when the node is active than in sleeping mode. When the energy of a sensor is totally exhausted, the sensor dies. Its death modifies the simplicial complex representative of the coverage by removing the corresponding vertex and the incident edges, triangles, and tetrahedrons. Consequently, we use the procedures described in Section 4 to update the discrete vector field. Moreover, the improvement proposed in the Section 4.2 is also implemented.

Using the discrete vector field underlying the complex *X* maintained by the updating algorithms, the sensor network nodes can compute and track a generator of $H_{2}\left(X,F\right)$, the sensors selected from which are activated and the others are put into sleeping mode.

The initial state of the network is displayed in the subfigure (a) of Figure 11. The entire area is well covered, and some places are covered more than once. Only 102 of the 211 sensors are activated in order to ensure this coverage.

The node 186, circled in Figure 11a, disappears because of its energy exhaustion. The discrete vector field updates as the resulting complex in Figure 11b shows, and activates only the node 120 to fill the hole created by the death of node 186. Due to the stability of our method, the other nodes are not affected by this modification.

Sometimes, the magnitude of the modification is important because the solution requires the activation of several sensors. For example, between states of Figure 11c,d, the extinction of the node 123, circled on the left, should be balanced by the activation of nodes 95 and 108. However, the activation of the latter node is redundant with the node 115, which is now put into sleeping mode. In this case, four nodes are involved in the correction of the coverage, unlike most cases where the activation of a single neighbor node is sufficient.

After the loss of several units, the network is no longer able to assure the coverage of the area. In Figure 11e, the extinction of the node 42 creates a hole in the coverage. Hopefully, this hole is detected by the vector field because of the presence of a critical edge that connects nodes 13 and 14. Being critical, this edge indicates that the group $H_{1}\left(X\right)$ is not trivial, and, following the edge, it is possible to locate the hole in the network.

However, in this case, even if the network does not cover the area, our algorithm activates only a limited number of sensors covering the rest of the surveillance area. The same phenomenon is observed following the extinction of the node 67, as shown in Figure 11f, which can also detect and locate the second hole in the coverage. As a caveat, this phenomenon was discovered by experimentation and is not theoretically founded: as $H_{1}\left(X\right)$ is not trivial, $H_{2}\left(X,F\right)$ is trivial and thus no selection could be done. However, the discrete vector field still exists and provides a flow that is followed by the selecting algorithm described below resulting in the last two subfigures.

### 6.2. Relative Homology and Morse Theory

Our algorithm needs a small modification to compute the relative entropy $H_{2}\left(X,F\right)$: an artificial 2-cell, named π is introduced, whose boundary is *F*, i.e., ∂π=F. The introduction of the cell π brings the isomorphism $H_{2}\left(X,F\right)≃H_{2}\left(X\cup \pi \right),$ which converts relative homology group computation into an absolute homology group computation that our algorithm can tackle.

When the sensors cover the area, the group $H_{2}\left(X\cup \pi \right)$ is non-trivial. It has only one generator and, according to the Morse theory, a representative is given by the fixed point $\Phi^{\infty }\left(\pi \right)$ of the flow defined by the vector field. From [24], if the collection of the vertices of the triangles of this representative are active, the area is fully covered by the network. Even if the set of active nodes ensures the coverage, its size is not necessarily minimal. However, in practice, the solutions are good enough.

We present below two methods to compute the fixed point of the flow and quickly. The first method is exact, but involve some implementation constraints that do not scale well with the size of the sensor network. The second method resolves this problem but with a small loss of optimality.

### 6.3. The First Activation Method

The first method is a direct translation of the definition of the fixed point $\Phi^{\infty }\left(\pi \right)$ into an algorithm. However, the cell π is an artifact for the calculation: we know its boundary, *F*, but the cell ϕ itself is not managed by any node. Including this cell π into the data structure managing the simplicial complex is not recommended: it is necessary to avoid a direct reference to π in the algorithm.

However, as π is a cell in the extended complex X∪π and it is absent from the data structure, this cell will be obviously labeled as a critical cell by the discrete vector field. Because of this criticality and using the definition of the flow, Φ(π)=π+V∂π+∂Vπ=π+VF.

The first method is summarized by Algorithm 2, which is the computation of the iteration $\Phi^{k}\left(\pi +VF\right)$ until a fixed point is reached. According to the previous derivation, the iterations are done without reference to π since at each iteration $\Phi^{k}\left(\pi \right)$ is π+σ.

**Algorithm 2:** First method to compute the activated sensors.  
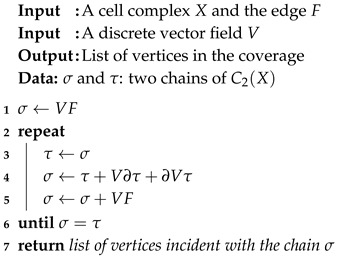


**Figure 12:**
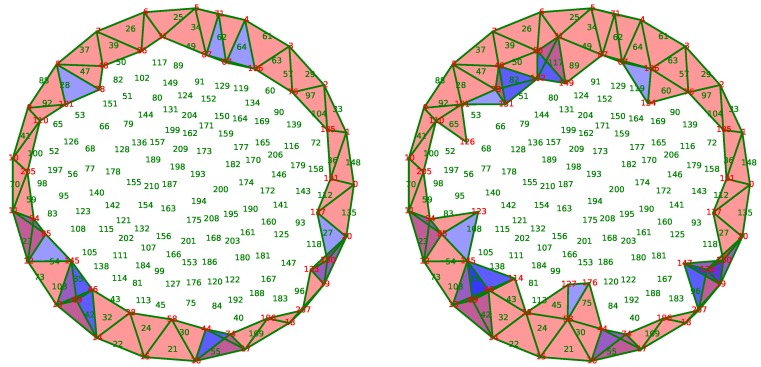
Steps of the first method.

Figure 12 shows one iteration of this algorithm in which the active nodes are in red, while the others are in green. The 2-chain σ in construction is in pink, and blue triangles indicate the triangles with three active edges but not in σ. The subfigure on the left represents the chain σ at the beginning and the one on the right represents the same chain at the end of the step. Intuitively, we can see that the algorithm constructs gradually a coverage hanging on the boundary *F* and following the direction of the vector field *V* to cover the whole space *X*.

This method has the advantage to find a generator of $H_{2}\left(X,F\right)$ exactly, but it requires coordination among the nodes: first of all, all sensors should be synchronized at the beginning of each iteration; secondly, the halting condition needs a synchronization between the nodes to decide if a fixed point is reached. These synchronizations among nodes will deteriorate the distribution of the algorithm since they require the establishment of a master node in the whole network. An operation that does not scale well with the size of the network.

### 6.4. The Second Activation Method

The second method addresses the disadvantages of the first method. However, the constraint of exact calculation should be released. The price is not too high because it does not break the guarantee of coverage. The drawback is that the number of necessary nodes may be somewhat larger than that suggested by the first method. In practice, the difference is often less than a few units.

The idea is to then extract incident vertices as with the first method without computing a generator $H_{2}\left(X,F\right)$. Instead, the idea is to work directly with the active vertices. To achieve this, we follow the boundary of the iterates $\partial \Phi^{k}\left(\pi \right)$ and all the vertices on this boundary is activated. This process is easy because of the commutativity between the operators Φ and *∂*, that is ∂Φ=Φ∂: following $\partial \Phi^{k}\left(\pi \right)$ is equivalent to follow $\Phi^{k}\left(\partial \pi \right)=\Phi^{k}\left(F\right)$. As an aside, the cell π is not needed anymore.

Algorithm 3 contains the whole construction in the form of a list of statements. The set of activated nodes is built during the iterations and not at the end of the convergence. Moreover, the halting condition is easy to test because it corresponds to the nullity of the chain σ.

**Algorithm 3:** Second method to compute the activated sensors.  
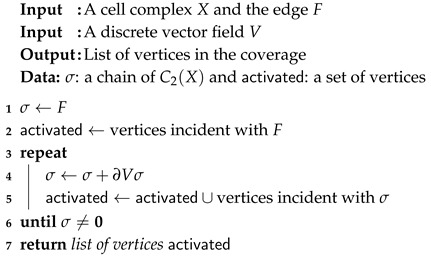


**Figure 13:**
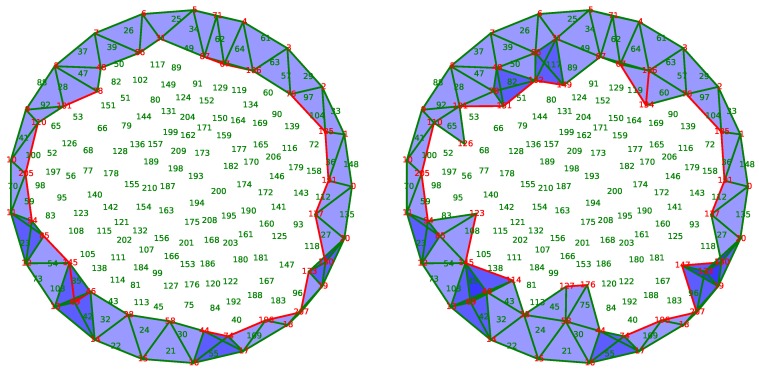
Steps of the second method.

Figure 13 shows an iteration of the second method. The chain σ is now a 1-chain, represented by the red edges, contrary to the 2-chain of the first method. Note that there is a great similarity between the two methods: the edges of the 2-chain σ of the first method are almost the same as the 1-chain σ of the second method. It is the initial idea behind this method. Intuitively, the second method “turns on” the edges *F* and propagates the front of the fire according to the discrete vector field *V* until it disappears to cover the burnt area.

It should be noted that the second method can be easily implemented in a distributed way. Indeed, by linearity, it is not necessary to “turn on” all the edges of the fence *F*. The method may start by any edge one after another in any order. Moreover, all computations are localized and the flow computation is only the matter of some neighbor nodes. No coordination is thus needed. It means that the edges of *F* may start the algorithm locally and randomly without worrying about the other part of the network.

In the first method, depending on the vector field, a triangle may be added in the chain σ during an iteration and be removed later because it is visited twice by the flow and because, in the binary field, 2=0. However, with the same setting in the second method, the vertices of this triangle added to the list of activated nodes in line 5 cannot be removed afterward. Consequently, all vertices activated with the first method are also activated with the second method, but the vertices of triangles visited twice are also present with the second method.

## 7. Conclusions

Adaptive discrete vector fields provide a distributed method to compute the generators of the homology groups of a cell complex distributed in a network. These groups give powerful topological invariants that reflect the connectivity of the network.

In the solution presented in this paper, the construction of a distributed discrete vector field is done alongside the additions and the removals of cells in the complex. These methods follow dynamically the evolution of the network: the adaptive nature of the provided solution relies on the discrete vector field, which is a collection of local data managed locally. Thus, network changes affect only the part of the network where they occur.

Moreover, the primitive operations performed by our algorithms concerns only neighbor nodes and may be performed in parallel. Thus, as no global synchronization is necessary, our solution may be implemented in a distributed way and scales well with the size of the network.

The applications of our method to problems concerning the coverage and the selection of active sensors show the generality of our approach.

Sensor networks benefit from other applications of homology groups’ computation such as tracking [13] or network management [31]. Moreover, discrete vector fields are also useful within the sheaf approach applied to networks [32] as this theory appears to be well-suited for the integration of heterogeneous and non-localized data [33,34,35,36]. Our objective is to extend our solution in order to compute relevant information extracted from a sheaf in a distributed and adaptive way based on the work [37].
